# The impact of oral appliance therapy with moderate mandibular advancement on obstructive sleep apnea and upper airway volume

**DOI:** 10.1007/s11325-019-01914-3

**Published:** 2019-08-10

**Authors:** Riitta Pahkala, J. Seppä, R. Myllykangas, J. Tervaniemi, V. M. Vartiainen, A. L. Suominen, A. Muraja-Murro

**Affiliations:** 1 Department of Oral and Maxillofacial Diseases, Kuopio University Hospital, P.O. Box 100, 70029 Kuopio, Finland; 2 Head and Neck Center, Kuopio University Hospital, Kuopio, Finland; 3 Institute of Dentistry, Department of Medicine, Faculty of Health Sciences, University of Eastern Finland, Kuopio, Finland; 4 Department of Clinical Radiology, Kuopio University Hospital, Institute of Dentistry, Oral and Maxillofacial Radiology, University of Eastern Finland, Kuopio, Finland; 5 Department of Oral Public Health, Institute of Dentistry, University of Eastern Finland, Kuopio, Finland; 6 Department of Clinical Neurophysiology, Diagnostic Imaging Center, Kuopio University Hospital, Kuopio, Finland

**Keywords:** Obstructive sleep apnea, Mandibular advancement device, Apnea-hypopnea index, CBCT, Upper airway volume

## Abstract

**Purpose:**

To find out if a moderate protrusion with a mandibular advancement device (MAD) can significantly increase the upper airway volume and, further, what signs and symptoms of obstructive sleep apnea (OSA) can be improved by this maneuver.

**Methods:**

There were 58 adults diagnosed with OSA who were referred for MAD therapy. The mean apnea-hypopnea index (AHI) was 19.2 (SD 8.6). Five indicators of signs and symptoms of OSA (AHI, oxygen saturation, snoring, daytime sleepiness, and health-related quality of life) were evaluated at the baseline and after 6 months of MAD therapy. Nasal resistance and airway volume and cross-sectional areas with and without the MAD in situ were recorded. Based on AHI reduction, the treatment response was classified as complete, partial, or non-complete. Statistical analyses included the chi-square, *t* tests, Mann–Whitney U tests, and regression analyses (linear and logistic).

**Results:**

Twenty-three patients attained a complete response (residual AHI < 5 events/h) to MAD therapy. In 13 subjects, the response was partial, and in 9 patients, it was non-complete. The complete responders were significantly younger, and they had a deeper overbite than partial/non-complete responders. A convex profile associated positively, but a vertically restricted throat and increased lower facial height associated negatively with the increase in airway volume.

**Conclusions:**

Excellent MAD therapy outcomes were achieved in most patients. Only age and deep bite had some influence on AHI reduction, indicating multifactorial nature in the response to MAD therapy.

## Introduction

In obstructive sleep apnea syndrome (OSAS), there are recurrent episodes of partial or complete obstruction of the upper airway during sleep, resulting in sleep fragmentation and oxygen desaturations. OSA has been identified as an independent risk factor for cardiovascular diseases [1] and is associated with reduced quality of life, increased healthcare utilization, and mortality [2, 3]. Continuous positive airway pressure (CPAP) is the gold standard of treatment, but its effectiveness is limited by poor compliance and intolerance. Although oral devices are not as effective as CPAP therapy in reducing the apnea-hypopnea index (AHI), they offer an alternative for patients with mild to moderate OSA who are unable to tolerate CPAP therapy.

There is no consensus regarding the most effective mandibular advancement with oral devices. In an earlier review, it was suggested that the more the mandible is stretched forward, the more the AHI improves [4], while in the recent review and meta-analysis, it was concluded that the AHI improvement is not proportional to the increase of mandibular advancement [5]. Since OSA patients may suffer from different underlining pathophysiologies such as an upper airway anatomical abnormality, increased pharyngeal collapsibility, an overly sensitive ventilator control system, or a reduced arousal threshold, the inter-individual response to mandibular advancement device (MAD) therapy also varies [6]. The focus of this study was to find out if a moderate mandibular advancement (60%) with MAD can significantly increase the upper airway volume and, further, what signs and symptoms of OSA can be improved by this maneuver.

## Subjects and methods

### Subjects

**Fig. 1:**
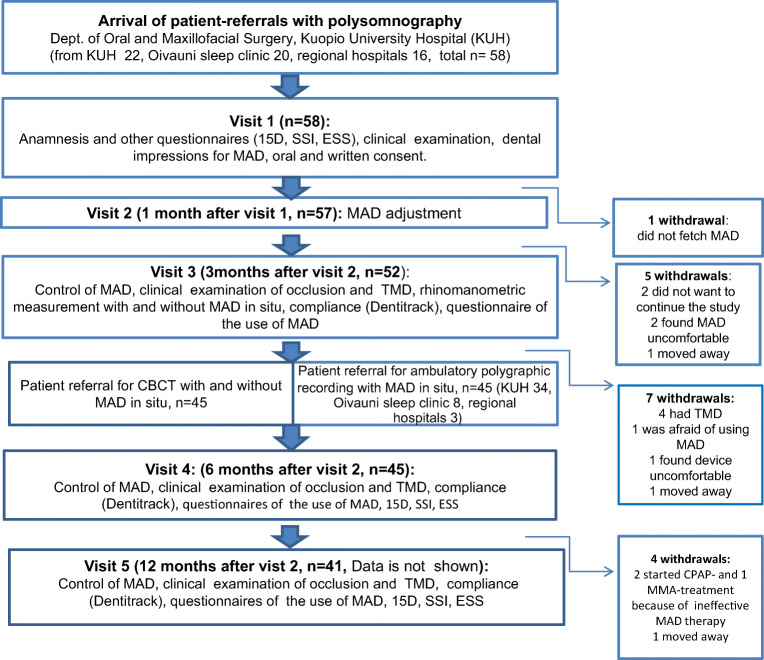
Study flowchart. CBCT, cone-beam computed tomography; ESS, Epworth sleepiness scale; MAD, mandibular advancement device; MMA, maxillomandibular advancement surgery; OSA, obstructive sleep apnea; SSI, snoring symptoms inventory; TMD, temporomandibular disorders; 15D, 15D health-related quality of life

All patients in this study were diagnosed as having OSA by ambulatory polygraphic recording (APR) and were referred by sleep medicine specialists for oral device treatment to the Oral and Maxillofacial Department, Kuopio University Hospital (KUH). Patients were consecutively recruited from the autumn of 2016 to the end of the year 2017. Subjects were enrolled if they were 18 years or older, their AHI was at least 10 events/h, their body mass index (BMI) was less than 35 kg/m^2^, and they had at least 5 teeth/jaw. This study protocol was approved by the Research Ethics Committee of the Hospital District of Northern Savo, Kuopio, Finland (7 February 2017; 80/2017). All patients provided written informed consent before participating in the study. The study flowchart is presented in Fig. 1.

### Methods

Five indicators of signs and symptoms of OSA (AHI, arterial oxyhemoglobin saturation (SaO_2_), snoring symptoms inventory (SSI), Epworth sleepiness scale (ESS), 15 D health-related quality of life (15D)) were evaluated in this study. Besides, changes in nasal resistance with the MAD in situ and upper airway volume and minimal cross-sectional areas with the MAD in situ were recorded. Data were also collected on age, gender, marital status, education, BMI, smoking, alcohol consumption, comorbidity, prescribed medication, and previous treatment of OSA.

### Ambulatory polygraphic recording

Nocturnal single-night ambulatory polygraphic recordings were conducted to diagnose OSA prior to this study and after 6 months with the MAD in situ. Home APRs were conducted by using either the Embletta (Embletta, Embla, Broomfield, CO, USA) or Nox A1 ambulatory polygraphic device (Nox Medical, Reykjavík, Iceland) with accepted guidelines for diagnosing OSA. Apneas and hypopneas were automatically scored (Remlogic, version 3.2, and Somnologica, version 3.2 software, Embla Co., Broomfield, CO, USA) and manually verified and edited. Apnea was defined as a cessation (≥ 90%) of airflow for ≥ 10 s. Determination of hypopneas was based on a decrease (≥ 30%) in airflow for ≥ 10 s and associated with oxygen desaturation ≥ 4. Trained physicians evaluated the recordings. The following parameters were assessed: AHI, supine AHI, SaO_2_, percentage of SaO_2_ below 90%, and proportion of sleep time spent snoring. Positional OSA was defined as a difference of 50% or more in apnea index between supine and non-supine sleep positions [7]. Based on the second APRs, patients were classified according to the treatment success of MAD therapy as follows: complete responders (residual AHI < 5 events/h), partial responders (residual AHI > 5 events/h, decrease in baseline AHI > 50%), and weak responders (residual AHI > 5 events/h, decrease in baseline AHI < 50%).

### Daytime sleepiness and quality-of-life measurements

In order to evaluate the discomfort in life due to OSA and snoring, the subjects were asked to fulfill ESS, SSI, and 15D questionnaires at the baseline and 6 and 12 months after MAD therapy. Daytime sleepiness was assessed using ESS score (range 0–24), with a score of 11 or more being indicative of excessive daytime sleepiness [8]. The SSI score was composed of two components: the social-work and the physical-embarrassment component including 25 symptoms (range 0–100), with a score of 0 corresponding to no problems with snoring [9]. Health-related quality of life among patients was assessed with the 15D questionnaire [10]. It contains 15 dimensions of life (questions), each having five different levels of functional status. These dimensions can be presented as a 15-dimensional profile or as a single index score. The score can vary between 0 and 1, a low score indicating more pathology than a high score.

### Clinical examination

Clinical data included occlusal findings (molar occlusion, overjet, overbite, crossbite, open bite, scissor bite, crowding, palatal morphology) assessed according to the modified method of Björk [11], examination of temporomandibular joints (TMJs), bite muscles, mandibular movements [12], craniofacial morphology (visual evaluation of the facial profile based on the angle formed by the soft tissue glabella, subnasale, and soft tissue pogonion, anteroposterior jaw relationships, and vertical proportions) [13, 14], and the Mallampati score [15]. The cephalometric measurements were not included in the original study protocol. Measurements were repeated at 3, 6, and 12 months after MAD therapy. All the examinations were based on routine orthodontic clinical examinations done by the same experienced orthodontist (RP). In our previous study [16], the accuracy of the evaluation of the facial profile by this method was good (kappa value 0.920 with the agreement of 93.3%).

### Rhinomanometric measurement

Rhinomanometry was performed 3 months after MAD therapy. An NR6-rhinomanometer (GM Instruments Ltd., Kilwinning, UK) was used to conduct the rhinomanometric recordings, in which total inspiratory nasal resistance was recorded at a radius of 150 Pa. Recordings were made in the supine position without MAD and MAD in situ. Nasal decongestion was not used.

### Upper airway imaging

Images were taken about 6 months after MAD therapy on a CBCT machine with Romexis software (Promax 3D Max: Planmeca, Helsinki, Finland). CBCT imaging was done in an upright position with the Frankfort horizontal line parallel to the floor. The patients were advised to keep their head in an upright position, hold light intercuspidation, and breathe normally but not to swallow or do any conscious movements during the imaging and measurement (13 s).

The airway analysis tool was used to define the portion of interest and to calculate the volume and cross-sectional areas of each section. The sections were determined using the boundaries described elsewhere [17]. The image orientation and selection of sensitive threshold values were conducted manually. There were two experienced oral radiologists who measured the parameters.

### Mandibular advancement device therapy

Patients were treated with the SomnoDent Flex (SomnoMed Ltd., Sydney, Australia) custom-made acrylic duo block titratable oral device. In this device, the upper and lower splints are connected by adjustable interlocking acrylic buccal extensions. In order to determine the desired amount of mandibular advancement, the inter-occlusal bite was registered with the SOMGauge bite registration device to obtain 60% of the maximal protrusion of the mandible.

### Statistical analysis

Statistical analyses were performed using the IBM SPSS statistics, version 22.0 (IBM Corp., Armonk, NY, USA). For the statistical analysis, partial and non-complete responders were combined. The chi-square test was used to analyze the differences in categorical variables between males and females and between complete and partial/non-complete responders. Fisher’s exact test was used when the numbers of subjects in some cells were small. The differences in continuous variables were analyzed using Student’s *t* test for normally distributed variables and the Mann–Whitney *U* test for variables with skewed distributions. Multivariate linear regression analysis was used to investigate the associations of age, gender, baseline and positional AHI, convex profile, extreme overjet, increased lower facial height, decreased palatal width, and vertically restricted throat with an increase in total airway volume. Furthermore, logistic regression analysis was used to study the association between treatment response dichotomized complete response vs. partial/non-complete response as a dependent variable and age, gender, BMI, convex profile, overjet, overbite, cross bite, increase in airway volume, and vertically restricted throat. The independent variables were added simultaneously in regression analyses and their choices were based on previous literature which suggests that they are related to sleep-disordered breathing and upper airway volume. Also, some of them contributed to the outcomes in bivariate analyses. Associations with *p* values of < 0.05 were considered statistically significant.

The intra- and inter-examiner reproducibility of CBCT measurements for airway volume and minimum cross-sectional areas were investigated by repeating measurements (*n* = 20) of those variables 2 weeks apart by two experienced oral radiologist using intra-class correlation coefficients (ICC) and its 95% confidence interval (CI). The intra-examiner repeatability of CBCT measurements varied from 0.945 (hypopharynx volume) to 0.996 (minimal cross-sectional area of the oropharynx), but it was only fair for the minimal cross-sectional area of the nasopharynx (0.387). The inter-examiner repeatability varied from 0.774 (minimal cross-sectional area of nasopharynx) to 0.995 (volume of oropharynx).

## Results

During the recruitment period, 39 males and 19 females diagnosed to have OSA were consecutively referred for MAD therapy. The mean AHI of the subjects was 19.2 (SD 8.6), with no gender difference (Table 1). The mean age of the subjects was 50.7 years (SD 11.9). On average, women were significantly older than men, were more often single, and were more highly educated than men. In half of the participants (49%), OSA was positional, and fifteen patients had tried CPAP therapy.

**Table 1:** Characteristics of subjects at the baseline and withdrawals during the first 6 months

	Male (*n* = 39)	Female (*n* = 19)	*p*	Total (*n* = 58)	Withdrawals (*n* = 13)	*p*
Age (years),^a^ mean (SD)	48.2 (12.7)	55.8 (7.1)	0.018	50.7 (11.9)	50.8 (11.5)	0.957
BMI,^a^ mean (SD)	27.7 (3.8)	28.1 (3.8)	0.721	27.9 (3.7)	29.2 (5.6)	0.198
AHI, events/h,^a^ mean (SD)	19.0 (8.8)	19.6 (8.4)	0.813	19.2 (8.6)	15.2 (7.4)	0.208
Data based on a questionnaire						
Marital status, (*n* = 47), single, *n* (%)	2/30 (6.7)	8/17 (47.0)	0.001	10 (21.3)	2/8 (25.0)	0.550
Education (*n* = 40), polytechnic/univ., *n* (%)	11/24 (45.8)	15/16 (93.8)	0.002	26 (65.0)	5/8 (62.5)	0.529
Daily smoking (*n* = 47), *n* (%)	3/30 (10.0)	3/17 (17.6)	0.450	6 (12.8)	1/8 (12.5)	0.733
Weekly alcohol consumption (*n* = 47), *n* (%)	16/30 (53.3)	7/17 (41.2)	0.423	23 (48.9)	4/8 (50.0)	0.625
Medical co-morbidities (*n* = 56)						
Hypertension, *n* (%)	11/37 (29.7)	8/19 (42.1)	0.354	19 (33.9)	2/11 (18.2)	0.193
Type 2 diabetes, *n* (%)	1/37 (2.7)	4/19 (21.0)	0.041	5 (8.9)	2/11 (18.2)	0.251
Mental disease/depression, *n* (%)	5/37 (13.5)	6/19 (31.2)	0.107	11 (19.6)	1/11 (9.1)	0.304
Prescribed medication (*n* = 56), *n* (%)	21/37 (56.8)	14/19 (73.7)	0.215	35 (62.5)	6/11 (54.5)	0.391

Data are numbers (percentages) and *p* values from the chi-square test or from Fisher’s exact test

^a^Data are means (standard deviations) and *p* values are from Student’s *t* test

More than half of the patients (*n* = 23) attained a complete response (AHI < 5/h) to MAD therapy; in 13 subjects, the response was partial, and in 9 patients, it was non-complete. The results showed that the complete responders were significantly younger, and they had a deeper overbite than partial/ non-complete responders (Table 2). MAD therapy significantly reduced AHI and increased SaO_2_ almost significantly (Table 3). Also snoring, the SSI score, and daytime sleepiness were significantly reduced. The increases in total upper airway volume and oropharyngeal volume with MAD in situ were both significant. Also the minimum cross-sectional areas increased significantly in the nasopharynx, oropharynx, and hypopharynx. Further, the decrease in nasal resistance with MAD in situ was significant.

**Table 2:** Characteristics of subjects according to treatment response to mandibular advancement device therapy

	Complete responders *n* = 23	Partial/non-complete responders *n* = 22	*p*
Characteristics of subjects			
Gender			
Females, *n* = 13, *n* (%)	7 (54)	6 (46)	0.815
Males, *n* = 32, *n* (%)	16 (50)	16 (50)	
Age (years),^a^ mean (SD)	46.7 (13.3)	54.8 (8.6)	0.019
BMI^a^, mean (SD)	28.0 (3.3)	27.1 (2.8)	0.388
Ambulatory polygraphic recordings			
Baseline AHI,^a^ mean (SD)	18.4 (9.1)	21.4 (8.2)	0.252
Positional AHI, *n* (%)	14 (61)	8 (36)	0.226
Oxygen saturation,^a^ mean (SD)	94.0 (1.5)	93.7 (1.5)	0.470
AHI after treatment,^a^ mean (SD)	2.6 (1.7)	10.1 (4.7)	< 0.001
Dentofacial and pharyngeal features			
Overjet (mm),^a^ mean (SD)	3.1 (1.5)	2.3 (1.8)	0.113
Overbite (mm),^a^ mean (SD)	4.0 (1.8)	2.8 (2.0)	0.044
Crossbite, *n* (%)	2 (9)	5 (23)	0.188
Convex profile,^c^ *n* (%)	10 (44)	7 (32)	0.420
Mandibular retrusion,^c^ *n* (%)	11 (48)	5 (23)	0.058
Vertically restricted throat,^b^ *n* (%)	9 (39)	11 (50)	0.758
Mandibular protrusion with MAD (mm), (SD)	5.9 (1.2)	5.6 (1.5)	0.344
Changes in upper airway space			
Increase in airway vol. (cm^3^),^a^ mean (SD)	7.0 (5.8)	4.2 (5.8)	0.207
Increase in min. oropharyngeal area (mm^2^),^a^ mean (SD)	201 (199)	101 (257)	0.250
Nasal resistance			
Nasal resistance without MAD in situ,^a^ mean (SD)	0.37 (0.10)	0.44 (0.22)	0.318
Nasal resistance with MAD in situ,^a^ mean (SD)	0.33 (0.08)	0.37 (0.15)	0.381
Changes in symptoms of OSA			
Decrease in the SSI score,^a^ mean (SD)	5.7 (11.1)	15.6 (19.3)	0.149
Decrease in the ESS score,^a^ mean (SD)	0.9 (2.8)	2.1 (2.9)	0.314

Data are numbers (percentages) and *p* values are from the chi-square test or from Fisher’s exact test

^a^Data are means (standard deviations) and *p* values are from Student’s *t* test

^b^Defined as Mallampati et al. class 4

^c^Defined by the visual evaluation of the facial profile

**Table 3:** Baseline determinants and treatment outcomes 6 months after MAD therapy, and upper pharyngeal airway patency and nasal resistance with and without MAD in situ

Variables	Baseline data/data without MAD in situ mean (SD)	Data after 6 months therapy /MAD in situ mean (SD)	p*
Ambulatory polygraphic recordings (*n* = 45)			
AHI, events/h	19.9 (8.7)	6.4 (5.2)	< 0.001
Positional AHI, events/h	34.0 (16.3)	11.7 (8.6)	< 0.001
Mean oxygen saturation (%)	93.8 (1.5)	94.2 (1.5)	0.052
Percent of time with			
Oxygen saturation lower than 90%	5.4 (12.5)	2.8 (10.2)	0.011
Snoring (%)	31.2 (21.6)	19.7 (15.6)	0.015
Symptoms of obstructive sleep apnea			
Snoring symptoms inventory score (*n* = 23)	37.0 (20.6)	27.5 (19.5)	0.009
Social work score	8.1 (10.8)	4.2 (7.7)	0.046
Physical embarrassment score	7.5 (5.5)	5.7 (6.1)	0.047
15D – quality of life score (*n* = 20)	0.877 (0.094)	0.892 (0.094)	0.194
Epworth sleepiness scale (*n* = 27)	6.2 (4.1)	4.9 (3.9)	0.038
Upper airway space (*n* = 34)			
Nasopharynx			
Volume (cm^3^)	8.89 (3.1)	8.74 (2.9)	0.541
Minimum cross-sectional area (mm^2^)	510 (313)	626 (364)	0.027
Oropharynx			
Volume (cm^3^)	14.7 (8.1)	19.0 (9.5)	< 0.001
Minimum cross-sectional area (mm^2^)	459 (334)	590 (378)	0.002
Hypopharynx			
Volume (cm^3^)	5.0 (2.3)	5.4 (3.3)	0.144
Minimum cross-sectional area (mm^2^)	673 (375)	767 (411)	0.023
Total pharyngeal airway volume (cm^3^)	27.3 (13.4)	32.3 (13.8)	< 0.001
Nasal resistance (*n* = 22)			
Mean total nasal inspiratory resistance (Pa/cm^3^/s)	0.40 (0.15)	0.35 (0.11)	< 0.001

*By paired *t* test

In multivariate linear regression analyses, a convex profile associated positively but a vertically restricted throat and increased lower facial height associated negatively with the increase in upper airway volume (Table 4). No factor that influenced the response of MAD therapy on AHI was identified by logistic regression model using the criterion of complete response as a dependent variable (data not shown).

**Table 4:** Predictive factors for an increase in total upper airway volume with MAD in situ (*n* = 34). Data are from multivariate linear regression model; the effects of age and gender were considered

	Beta	95% Cl	*p* value
Male gender	0.248	− 1.32–7.80	0.153
Age (years)	0.242	− 0.11–0.38	0.281
AHI at baseline, events/h	0.363	− 0.01–0.48	0.054
Positional AHI	− 0.241	− 7.59–1.68	0.197
Convex profile^a^	0.634	2.25–13.56	0.009
Extreme overjet (> 5 mm)	− 0.19	− 12.44–3.31	0.240
Increased lower facial height	− 0.338	− 16.05 to − 0.09	0.048
Decreased palatal width	0.174	− 1.43–4.21	0.314
Vertically restricted throat^b^	− 0.494	− 10.81 to − 1.32	0.015

^a^Defined by the visual evaluation of the facial profile

^b^Defined as Mallampati et al. class 4

## Discussion

In the present study, convex facial profile proved to be a predictive factor for an improvement in the total pharyngeal airway volume with MAD therapy, whereas increased lower facial height and a vertically restricted throat seemed to worsen treatment outcome. In subjects with a convex profile, the overjet was significantly larger and the mean mandibular protrusion capacity was greater than in those with a straight or concave profile (Pahkala et al. MS). Thus, in subjects with a convex profile, the mandibular advancement (60%) in MAD was also larger, further increasing the pharyngeal airway volume. Interestingly, although not significantly related, the association between an extreme overjet and the increase in airway volume was negative. This tendency is parallel to previous 2D cephalometric and 3D volumetric studies on jaw orthopedic patients with mandibular and maxillomandibular advancements [18, 19], indicating that there is a certain limit after which no further improvement in airway space can be achieved after increasing the mandibular advancement. If this were also the case in MAD therapy, then a moderate mandibular advancement with MAD should be recommended. In addition, increased lower facial height seemed to limit the airway volume increase with treatment. This kind of craniofacial anatomy predisposes the mandible to rotate clockwise with the MAD in situ, thus reducing the retrolingual pharyngeal space.

Clear improvement in AHI by MAD therapy has been found to correlate to a retrognathic mandible [20], large upper inter-molar width [21], and wide lower inter-canine width [22]. In the present report complete responders had significantly deeper (over)bite and they tended to have more often mandibular retrusion than partial /non-complete responders. Our findings indicate that in subjects with a deep bite the intermaxillary space for the tongue in the oral cavity is reduced, which is then normalized by mandibular advancement with MAD. Thus our finding strengthens the previous suggestion [22] that the ratio between the tongue and bony enclosure size may be a predictive factor for the response of MAD therapy. Besides changing the position of the jaw and tongue MAD therapy is likely to increase the m. genioglossus tone and thus reduce the collapsibility of the upper airways. Furthermore, it has been found that in patients with Mallampati classes 3 and 4 the incidence of sleep apnea is high [23]. In the linear regression analysis, a vertically restricted throat (Mallampati class 4) was negatively related to an improvement in upper airway volume, indicating that soft tissue enlargement in the throat impairs the treatment response to MAD therapy.

Altogether low-dose CBCT proved to be an ideal diagnostic aid to study the pharyngeal airway in 3D. In the present study, the validity of CBCT to assess the upper airway space was good since both intra- and inter-examiner consistencies were high and acceptable. Generally, an increase in the airway volume increases airway flow and thus is likely to improve AHI and oxygen saturation level. Our results confirm this perception. The most critical site that causes maximum resistance to airflow is the smallest cross-section area of the airway. In our study, the smallest mean cross-sectional area in the pharynx was in the oropharyngeal region, which was increased 202 mm^2^ by MAD in situ in complete responders and half of that in partial/non-complete responders.

Concerning the effect of age on MAD treatment success, previous studies are contradictory [21, 22, 24]. In the present sample, complete responders were significantly younger than partial/non-complete responders. As observed previously, natural pharyngeal structures continue to grow until 13 years of age [25] and begin to narrow from about 20 until 50 years most likely because the soft palate becomes longer and thicker [26]. This narrowing might also be due to weight gain and an increase in the pharyngeal fat pad.

In normal subjects, upper airway resistance is lower during sleep when breathing through the nose. With nasal obstruction nose breathing switches to mouth breathing which is associated with increased propensity to OSA [27]. Nasal resistance is suggested to be elevated in patients with OSA [28, 29], but it is not related to the severity of OSA [30], or to AHI [31]. It also seems that high nasal resistance is a result rather than a cause of OSA, since nasal resistance decreases after tonsillectomy and AHI reduction [32]. Our result is parallel to this finding since nasal resistance was decreased significantly by upper airway enlargement with MAD in situ.

**Fig. 2:**
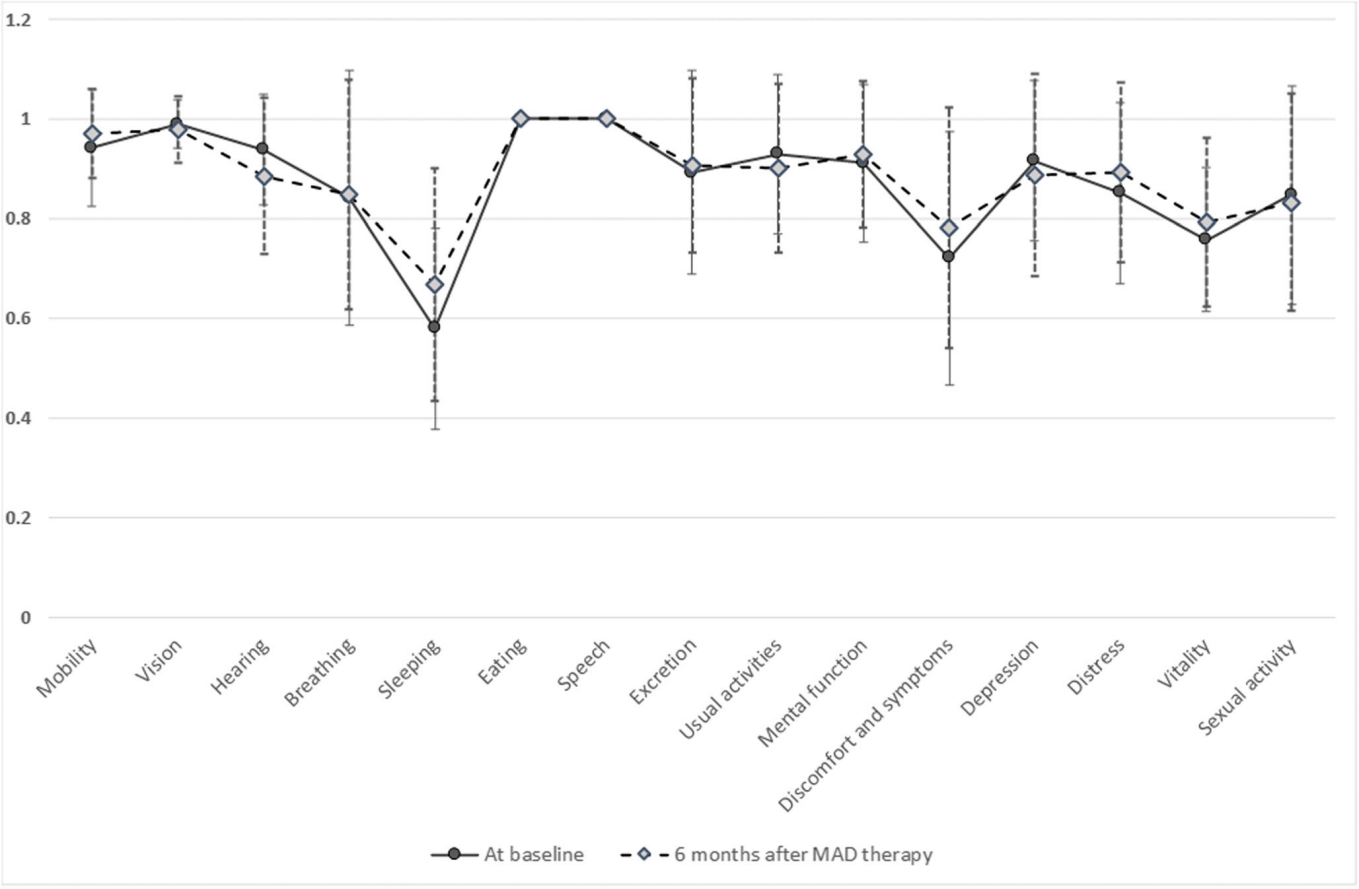
15D health-related quality of life dimension scores at the baseline and 6 months after MAD therapy. Move, mobility; see, vision; hear, hearing; sleep, sleeping; eat, eating; speech, speech; excret, excretion; uact, usual activities; mental, mental function; disco, discomfort and symptoms; depr, depression; distr, distress; vital, vitality; sex, sexual activity

Our results showed that the prevalence of snoring was significantly reduced by MAD therapy. However, some patients still complained about persistent snoring, which may partly be related to the device design allowing mouth opening during sleep [33]. Interestingly, although in partial/non-complete responders AHI decreased less but the SSI score more than in complete responders, the adherence to MAD therapy seemed to be equal in the groups (Pahkala et al. MS). This underlines patients’ interest to reduce snoring in order to eliminate disturbances in everyday life. In the present study, the mean ESS scores both at the baseline and 6 months after MAD therapy were within the normal limit, which agrees the suggestion that mild to moderate OSA causes less pronounced daytime sleepiness than previously assumed [33]. Concerning the 15D quality of life, certain dimension scores such as “sleeping,” “discomfort and symptoms,” “psychological distress,” and “vitality,” however, were lower at the baseline than after using MAD for 6 months, indicating some minor improvements in those dimensions of life (Fig. 2).

There is a variety of definitions of good MAD therapy response, and a lack of consensus around this issue makes the comparison between studies difficult. Although MAD therapy on OSA has not been reported to be as effective as that of CPAP treatment, we suggest that the criteria concerning good MAD therapy outcome should be more ambitious with its goal being parallel to that of CPAP treatment. In previous studies, the complete response to MAD therapy has been reported to vary from 29 to 71% (mean 48%) [34]. In the present study, AHI normalized in 51% of the patients, indicating good treatment response.

All the patients in this sample were consecutively referred for MAD treatment from the catchment area of KUH, and thus, the referral bias is unlikely. The percentage of withdrawals (29%) in this study was in line with similar studies and is most likely due to consecutive patient recruitment protocol. Certain limitations should be noted when interpreting the results. In this study, the ambulatory polygraphic recordings were performed mainly in KUH, and also in other institutes. However, all analyses were made according to the standard AASM respiratory rules [35]. Rule 4A was used for scoring the hypopnea events. Furthermore, CBCT imaging was done in an upright position with the Frankfort horizontal line parallel to the floor. In order to capture the true upper airway dimensions and volume in an upright position, the patients should have been positioned in the natural head position since manual placing of the patient in a defined position has an effect on the airway parameters. However, neither of these upright positions during imaging reflects the true anatomy while sleeping in bed, and thus, the methodological bias is likely. In the present study, the CBCT images with and without MAD were taken consecutively thus eliminating the possible interference of seasonal change or different patient positioning during CBCT scannings.

Regarding the questionnaires, it should be noticed that not all the patients returned every questionnaire despite previously given instructions. Mainly the 15D questionnaire was omitted. Also some patients did not want the CBCT images to be taken, and in some patients, the rhinomanometric measurements failed due to nasal obstruction.

In summary, this study shows that even excellent treatment outcomes among OSA patients can be achieved with moderate mandibular advancement using an oral device. With this protocol, also the upper airway space improves remarkably. We also presume that patients’ adherence is good and that there are few side effects such as temporomandibular disorders and occlusal changes with this treatment protocol. Our further studies will focus on those issues.

## Conclusions

Excellent MAD therapy outcomes were achieved in most OSA patients with moderate mandibular advancement. A convex profile proved to be a predictive factor for a greater increase in pharyngeal airway volume, while increased lower facial height and a vertically restricted throat limited the airway volume increase. Despite some differences in the variables between complete and partial/non-complete responders, only age and deep bite seemed to have some influence on the effectiveness of MAD therapy. This indicates a multifactorial nature both in the pathophysiology of OSA and in the response to MAD therapy.
